# Exploring the facilitators and barriers to addressing social media’s impact on anxiety within primary care: a qualitative study

**DOI:** 10.3399/BJGPO.2023.0190

**Published:** 2024-05-29

**Authors:** Ailin Anto, Rafey Omar Asif, Arunima Basu, Dylan Kanapathipillai, Haadi Salam, Rania Selim, Jahed Zaman, Andreas Benedikt Eisingerich

**Affiliations:** 1 Institution: Faculty of Medicine, Imperial College London, London, UK; 2 Institution: Faculty of Life Sciences and Medicine, King's College London, London, UK; 3 Institution: Imperial College Business School, Imperial College London, London, UK

**Keywords:** anxiety, social media, primary health care, general practitioners

## Abstract

**Background:**

Several researchers and policymakers have acknowledged the alarming association between social media (SM) usage and anxiety symptoms in young adults. While primary care holds a crucial role in the improvement of health outcomes for those presenting with anxiety, there has been no research on GPs’ perceptions of the impact of SM on anxiety. Furthermore, there has been little discussion of SM as a risk factor in anxiety-related consultations. This study is the first to use empirical research to inform how primary care can adapt to address SM’s impact on anxiety within young adults.

**Aim:**

To identify the facilitators and barriers within primary care to addressing SM’s impact on anxiety among young adults.

**Design & setting:**

A qualitative study of GPs in the UK.

**Method:**

Following an exploratory pilot interview, semi-structured interviews with GPs (*n* = 7) were transcribed and thematically analysed, following an inductive approach.

**Results:**

The following six facilitators were identified: a framework to facilitate discussion; open GP attitudes; GP training; referral pathways; larger stakeholder influence; and young adult education of social media’s impact on anxiety. The following three barriers were identified: a lack of GP awareness of SM’s impact on anxiety; cautious GP attitudes; and increased pressure on the health service.

**Conclusion:**

This qualitative study revealed a diversity of perceptions, and these novel findings are instructive in the adaptation of primary care services to meet the current mental health needs of young adults, as well as better assisting GPs in engaging in these conversations, especially within university practice.

## How this fits in

The relationship between social media (SM) and the mental health of young people is intricate, and anxiety is one mental health condition associated with SM use. Despite this impact, there is currently a lack of guidance for practitioners, particularly in primary care, to effectively address anxiety related to SM use in young individuals. This study aims to identify factors essential for addressing this issue in primary care and provides a consultation framework to assist GPs in guiding conversations about patients' SM use.

## Introduction

Social media (SM) considerable influence young adults' lives and has various implications for anxiety.^1^ Understanding anxiety’s contributing factors is crucial for effective management.^2,3^ Empirical work suggests a positive association between SM and anxiety.^4–6^ The type of SM activity emerges as an important factor in inducing anxiety.^7^ For example, comparing oneself to content on SM, aligned with Festinger’s social comparison theory, is thought to be a key driver of anxiety.^8–10^ Other mechanisms include fear of missing out (FOMO), cyberbullying, stress, envy, and self-esteem, and potentially others not yet explored.^7,11,12^ Understanding these mechanisms is crucial for clinicians aiming to minimise the impact of SM on anxiety.

GPs serve as one of the first points of contact for young adults seeking help with anxiety.^13^ With 90% of mental health conditions managed in primary care,^14^ a holistic approach is essential to identify key anxiety drivers, including SM. Addressing SM’s impact on anxiety in primary care enables GPs to adopt a patient-centred approach, aligning with the evolving social climate.^15,16^

Despite the HEADS4 tool recognising SM’s significance in adolescent mental health,^17^ current National Institute for Health and Care Excellence (NICE) guidelines and mental health consultation toolkits for primary care lack specific identification of SM as an etiological factor.^18,19^ The lack of clinical and psychosocial research into the methods of addressing SM’s effect within primary care, specifically in young adults, forms one of the key rationales of this study. This study aims to identify facilitators and barriers in addressing SM’s impact within primary care. Furthermore, it aims to equip GPs with the ability to explore otherwise unfamiliar terrain, allowing for a greater societal impact by improving patient outcomes, especially within university practices.

### Aims and objectives

To identify the facilitators and barriers within primary care to addressing SM’s impact on anxiety among young adults.

## Method

### Design

A qualitative study was undertaken using semi-structured interviews and an inductive approach. This study conformed to the Standards for Reporting Qualitative Research framework.^20^

### Recruitment and sampling

GPs from different practices across the UK were recruited through convenience, snowball sampling, which took place in two phases: induction of first-line GP contacts; and recruitment of further GPs through first-line GPs. Non-UK GPs and trainees were excluded. Written and verbal informed consent was gained before the interview. Concurrent thematic analysis was carried out on interview data and recruitment was suspended at the point of data saturation.

### Data collection

Before data collection, one exploratory pilot interview was conducted by two researchers and one participant, allowing for analysis regarding interview length, style of question, and questioning technique.

Interviews were conducted remotely by one principal interviewer and one scribe (AA or AB). All interviews were transcribed verbatim and lasted 30–70 minutes in length. The semi-structured interview guide (Box 1) was initially informed by the aims and objectives of the project, and was adapted iteratively as data generation and analysis progressed.

Box 1 **Semi-structured interview guide for face-to-face individual interviews**

**Views on social media and wellbeing of patients**

What are your views on social media usage and wellbeing?

What are your views on social media usage and anxiety?

Do you feel that your patients would benefit from being asked questions about social media during a mental health consultation?

What are your general views on asking questions about social media?

**Future resources to raise awareness of problematic social media usage**

What would be beneficial for GPs to increase their awareness of problematic social media usage on mental health and help them to navigate conversations about social media during mental health conversation?

Do you know of any techniques or resources to tackle problematic social media usage?

If you were to identify social media as a significant contributor to one's anxiety, would this change the management or advice of the patient? Why?

What kind of changes would be made or what resources would you provide the patient with?

What would be the barriers to implementing such resources?

### Data analysis

#### Thematic analysis

Braun and Clarke’s six-step framework and Gioia *et al*’s approach were used to analyse interview data.^21,22^ Three researchers (HS, RS, and JZ) conducted thematic analysis for consensual validation. An iterative method was employed, refining codes and identifying thematic patterns throughout data collection and coding. In phases 1 and 2, initial concepts were extracted from independent reviews of transcripts, forming first-order codes. Phase 3 organised first-order codes into sub-themes and second-order themes. In Phase 4, themes were reviewed for external heterogeneity and internal homogeneity. In phase 5, instructive themes were defined, and the entire dataset was revisited for accuracy. Phase 6 involved reporting findings through an analytical narrative approach with examples from interview data.

#### Research trustworthiness

As the researchers have prior SM experiences, potential pre-existing assumptions about the link between SM and anxiety were acknowledged.^23^ The researchers, diverse in genders and ethnicities, were not GPs, minimising potential assumptions about GP opinions. The team conducted workshops with young adults to explore SM anxiety management and ways GPs could provide support. Reflexivity was emphasised, prompting researchers to question their own beliefs.^24^ To minimise bias, interviews were conducted by two researchers.^25^ Subsequent data analysis involved a dedicated team, achieving triangulation through independent extraction of first-order themes, integration of findings, and resolution of discrepancies by a third researcher. The entire research team comprehensively analysed the dataset to ensure investigator triangulation, acknowledging the interpretive nature of the research topic.^26^ Member checking verified interview transcript accuracy.

## Results

Seven GPs were recruited from across the UK as part of this study. All seven interviews took place online using Microsoft Teams.

### Demographics

Out of the seven GPs interviewed, five were male (71.4%) and two were female (28.6%). The ages of the GPs ranged from 30–57 years, with the mean age being 49.3 years (Table 1).

**Table 1.** GP demographics. A table showing the demographic characteristics of study participants

	Sex	Age, years	Ethnic group	Practice location
GP1	M	55	Other ethnic group	London
GP2	F	30	Asian	North West
GP3	M	42	Asian	London
GP4	M	57	White	West Midlands
GP5	M	55	Asian	London
GP6	F	57	Asian	East Midlands
GP7	M	46	Asian	North West

### Main themes from thematic analysis

#### Facilitators and barriers to addressing the impact of SM on young adults’ anxiety levels

**Figure 1.**
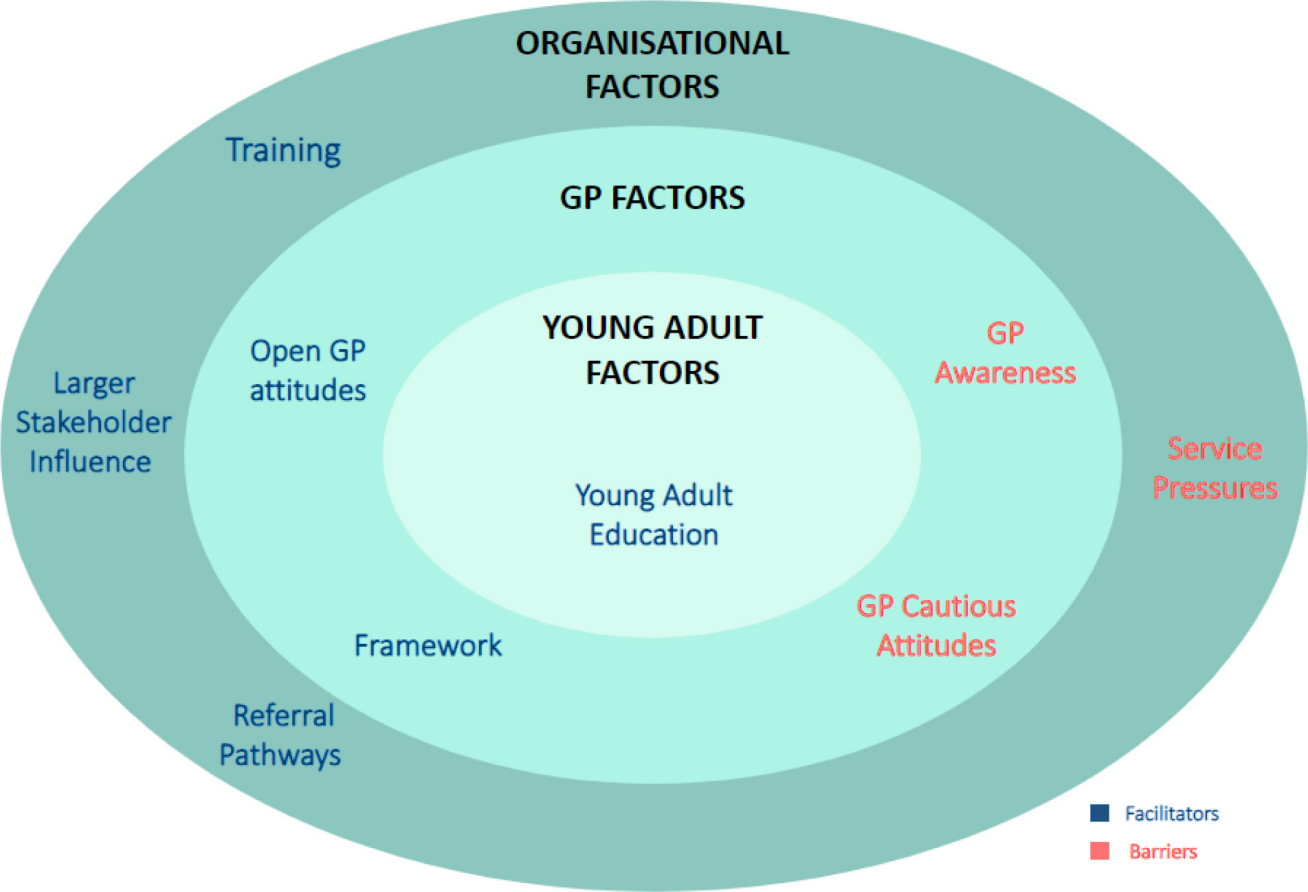
Facilitators and barriers diagram. Facilitators and barriers, derived from the interviews, categorised into three sections

In total six facilitators and three barriers to addressing the impact of SM on young adults’ anxiety levels were identified (Figure 1). The barriers and facilitators were organised into the following three second order themes: GP factors; organisational factors; and young adult factors.

- GP factors were any factors associated with GPs.
- Organisational factors highlighted factors associated with the structural characteristics of primary care.
- Young adult factors were deemed to be factors associated with young adults.

Researchers observed no pattern between GP characteristics and elicited second order themes and sub-themes.

### Barriers

The following three sub-themes (Table 2) emerged from the thematic analysis: lack of GP awareness; cautious GP attitudes; and service pressures.

**Table 2.** Second order and sub-themes for barriers in addressing social media’s impact on anxiety

Second order themes	Sub-themes
GP factors	GP awareness
	Cautious GP attitudes
Organisational factors	Service pressures

#### GP factors

##### Lack of awareness among GPs

A key barrier identified was the lack of awareness of SM’s impact on anxiety among GPs. This was primarily attributed to the generational gap between GPs and young adults, leaving them feeling unprepared to advise young adults on the matter:

*'I don’t really know much about it. I didn’t know that you could block people on Twitter. Or Instagram. Or whatever my daughter uses. It wouldn’t be me advising people about it!*' (GP6)

Some GPs revealed they were not regular users of SM and, thus, were unable to comprehend the context in which these platforms could affect their patient’s anxiety:

*'I just don’t know how those platforms work so what is the person doing if they say they’re doing this — I won’t understand the context.'* (GP7)

A few GPs explained that there was a lack of education around SM's impact on anxiety and mental health within their continued professional training as GPs:

*'... it’s not something* [SM] *that is in CPD* [continuing professional development]*, even, in our education as professionals.*' (GP5)

##### Cautious GP attitudes

Many GPs discussed their cautious attitude towards enquiring about SM’s impact on anxiety in mental health consultations with young adults, attributing this to the perceived intrusiveness of such a line of questioning:

*'... if you start going down that route, ... and perhaps the patient is defensive or feels that you're intruding, there’s always that possible danger, they may feel, what is the relevance of that question.*' (GP5)

GPs feared coming across as judgmental, with the danger of potentially causing a breakdown of communication:

*'People may feel embarrassed to talk about it, or they may feel inhibited to talk.*' (GP4)

*'... it sounds a bit too nosy. As a GP, I would say do you have you have a Twitter account, and they might start thinking I'm gonna stalk them.'* (GP2)

Additionally, a few GPs described the low priority that SM held for them within mental health consultations, as there were other lifestyle factors, such as alcohol consumption and smoking habits, among others, which held greater importance:

*'You need to know if they drink, have financial struggles, how everything is at home. There are lots of other questions to ask about ... my questioning regarding SM would come at the end. It is less of a priority for me.'* (GP6)

Lastly, a GP was sceptical of SM’s impact on mental health, as they believed that it was not a significant problem for most people. Moreover, they felt they had limited influence over a patient’s actions in this matter:

*'If I felt that asking about their SM would be the most helpful, I would. But I don’t feel that way ... I’m not convinced it is a huge problem for most so I don’t ask.*' (GP6)

### Organisational factors

#### Service pressures

Existing pressures on primary care services were cited as a prominent barrier with addressing SM’s impact on anxiety. GPs stressed that time constraints were a significant barrier they face during mental health consultations. GPs are required to take an in-depth history with multiple lines of questioning in a short timeframe, thus some doubted the value of enquiring about SM:

*'... we are so constricted with time, because we have very little time to assess patients.*' (GP4)

*'... the question is, is this going to be a valuable use of my time? Taking another what could take another five minutes?*' (GP5)

Some GPs also expressed reluctance in addressing SM’s impact on anxiety as it would just add to an already overwhelming workload:

*'... in a busy general practice, most of the time, GPs will think well, that’s just another thing you're asking me to do*.' (GP5)

*'... in my days off, I do a lot of paperwork during the weekdays ... on top of that, we've got, like our portfolios and things to do ... there’s plenty of things to get done.*' (GP3)

Additionally, many GPs emphasised the lack of funding for mental health care in the NHS, questioning the economic viability to sustainably tackle SM’s impact on anxiety in primary care:

*'I can't see any funding for that. No funding for mental health anyway.*' (GP5)

### Facilitators

The following six facilitators (Figure 1) were identified: open GP attitudes; a framework to facilitate discussion; GP training; referral pathways; larger stakeholder influence; and young adult education of SM’s impact on anxiety. These were again categorised into GP, organisational, and young adult factors (Table 3).

**Figure 2.**
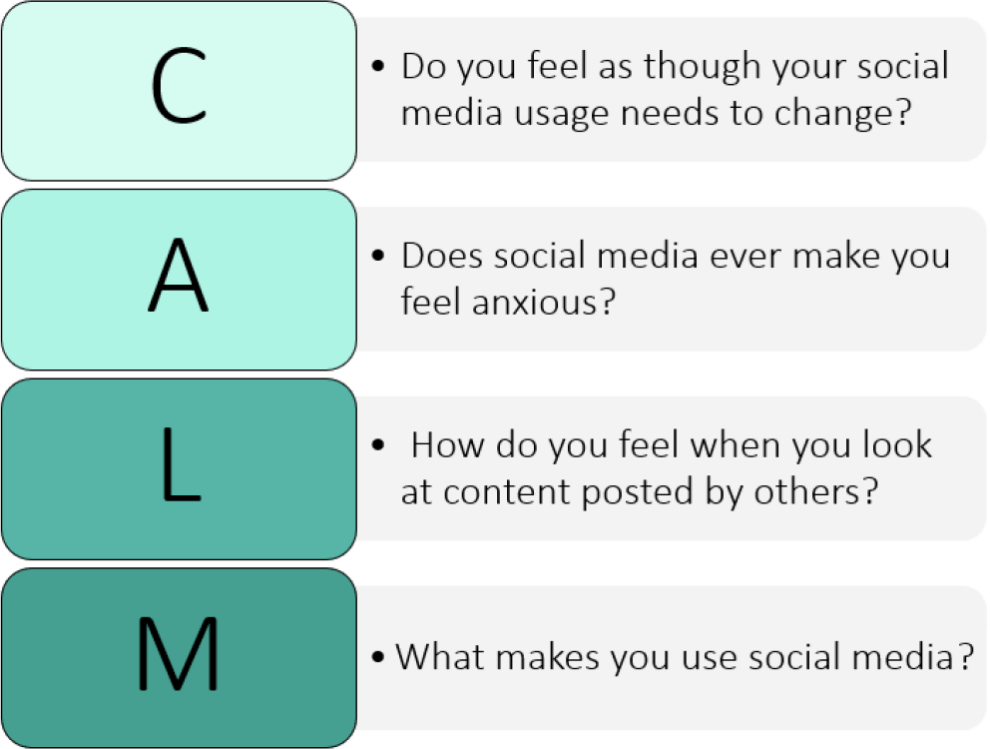
CALM framework outlining possible questions to explore social media’s impact on anxiety

**Table 3.** Second order and sub-themes for the facilitators in addressing social media’s impact on anxiety

**Second** order themes	Sub-themes
GP factors	Open GP attitudes
	Framework
Organisational factors	Training
	Referral pathways
	Larger stakeholder influence
Young adult factors	Young adult education

#### GP factors

##### Open GP attitudes

Some GPs referenced a need for greater awareness in how SM may be inducing anxiety in their patients. Only through this increased awareness can GP attitudes be transformed to address SM-induced anxiety:

*'... raise the awareness of the impact of SM in terms of mental health, both the positives and the negative. And I think raising that awareness to healthcare professionals, particularly GPs, so that they will consider it*.' (GP2)

GPs described the importance of an open attitude towards the acknowledgement of SM’s impact on patient anxiety levels, in addressing the issue. GPs appreciated that having had the impact of SM brought to their attention, they were more open to discussing the matter with patients than before:

*'I think we should focus more on this. And I've been discussing with my other colleagues in the university practice about this. It has highlighted SM use to us as well.*' (GP4)

Some GPs underscored the importance of the doctor–patient dynamic as a factor in facilitating constructive discussions around SM. They emphasised that cultivating a robust rapport between doctors and patients plays a crucial role in enhancing the comfort and efficacy of addressing patients' mental health concerns:

*'... if there is no good rapport between you and that patient, you're not* [going to] *get anything from them. But if you know them, you see them on a regular basis, you will manage to get everything and they will open to you, they will tell you everything.*' (GP1)

##### Framework

Many participants expressed a desire for a guiding framework in discussing SM use with patients. Most GPs agreed that, while their professional experiences could address uncertainties in these conversations, having a validated set of questions to guide discussions on SM use would be valuable:

*'... a structured questionnaire or structured way of approaching it ... that is resourced and effective.*' (GP5)

Furthermore, participants emphasised the potential benefits of integrating such a framework into existing NHS software systems to enhance motivation and ensure thorough exploration of SM’s impact:

*'... when you do the review, there’s a template, you follow on the system software,... where it would remind you to ask about that*.' (GP1)

GPs also suggested that incorporating questions about SM use, alongside other social history factors, could contribute to a more holistic understanding of a patient’s social context:

*'... we ask about sleep, about eating, about relationships, just different factors that affects mental health and anxiety, depression — all the triggers. So we could just add SM on it and see whether patients are affected by that in any way*.' (GP4)

### Organisational factors

#### Larger stakeholder influence

Many participants mentioned a need for increased awareness and action from key stakeholders that influence NHS policy and training, including General Medical Council (GMC), Health Education England (HEE), clinical commissioning groups (CCGs), and the Royal College of General Practitioners (RCGP):

*'.. as the issue becomes more and more topical ... I think, whether it is the RCGP or HEE, they need to think of what sort of training can we give primary care.*' (GP7)

It was noted that the implementation of a financial incentive, such as the Quality and Outcomes Framework (QOF), would encourage them to address the issue:

*'... let’s say it became obvious and there’s stacks of evidence, that one should then of course, that would end up on QOF. So that’s the incentive.*' (GP5)

For a community-driven approach, it was suggested that local mental health coordinators could be used as a means for raising awareness:

*'... it has to be done at grassroots level, where you have champions,... who are the Mental Health Leads.*' (GP2)

#### Training

A focus on SM within GP training was identified as a facilitating factor. It was suggested that such education should be implemented throughout medical training:

*'Get it as part of the mental health training that you get as a GP trainee, and that you get at medical school, so we start at the grassroots, and that will filter up*.' (GP5)

As part of this training, it was suggested that an online teaching module could be introduced on the eLearning For Healthcare (eLFH) platform, which is used for the continued training of clinicians:

*'... it’s a resource called elearning for health[care]... So if this is something that’s included, as part of that. I think it would reach everyone, because it’s used throughout the NHS.'* (GP3)

Moreover, GPs suggested extending such training to other allied health professionals in primary care:

*'... we have paramedics, we have pharmacists, we have physician’s associates, we have nurse associates — there is a whole wide range of clinicians now. So when you say GP training, it really needs to encompass all of the professionals in primary care.'* (GP7)

#### Referral pathways

GPs emphasised the importance of having appropriate mental health services to which they could refer patients with this problem, owing to limited capacity in primary care and the need to provide focused care for individuals:

*'The mental health services can take some of this workload, the student wellbeing they could have a dedicated unit that looks into students, young people affected by their SM. So that would be very helpful.*' (GP4)

The development of other community services, such as support groups, to offer targeted support, was suggested:

*'... developing pathways to help them but again, mental health is so overloaded. Alcoholics Anonymous for SM, is there actually a pathway you could send them to.'* (GP5)

Primary care network (PCN) mental health leads were highlighted to be potential advocates for change and to initiate local interventions to address the issue collaboratively.

*'So you would find the mental health lead for that network. And then the respective leads and each the practices, and maybe talk to them, and maybe a mental health workshop, where there is talking about the impact of digital interactions*.' (GP2)

### Young adult factors

#### Young adult education

Some GPs emphasised the importance of ensuring young adults are well informed about the potential impact of SM on anxiety. They highlighted the need for proactive education and suggested the creation of resources at individual practices to encourage young adults to gain understanding and motivate them to seek assistance:

*'Leaflet possibly to put in the surgery. Like if you use SM, you got abused, you think it’s affecting your mental health, speak to your doctor.'* (GP1)

A GP proposed the use of a questionnaire for individuals to self-assess their SM use and its impact on their anxiety. The aim would be to allow patients to reflect their own SM habits and if necessary, take steps to address identified issues independently:

*'... for patients, ... having a questionnaire or something to say is your SM use unhealthy or not, ... And if that you find there is an impact of it, you can just judge it for yourself, and consider the steps that are there on this website, to tackle it yourself.*' (GP3)

## Discussion

### Summary

The current study explored the barriers and facilitators in addressing the impact of SM on anxiety within primary care. The identified barriers included a lack of understanding among GPs, cautious attitudes toward SM’s impact on anxiety, and service pressures hindering exploration in time-constrained consultations. Facilitators comprised GPs' willingness to address the issue, a consultation framework to discuss SM’s impact, and wider stakeholder involvement to provide training and incentives. Training for the multidisciplinary team and established referral pathways in the community were recognised to lighten GPs' burden.

Regarding barriers, the study findings showed that GPs held varying degrees of scepticism regarding the impact that SM can have on anxiety, with some placing relatively low importance on the issue. However, with a lack of comprehensive models depicting the impact of SM on young adult anxiety and much of the evidence base demonstrating SM’s impact on anxiety being made up of cross-sectional studies, where causal relationships cannot always be determined, it is understandable that physicians have not yet been exposed to such information during their training, and lack understanding of the mediators and moderators at play.^4–6^ Forty per cent of GPs within the NHS are aged >50 years, a demographic with lower-than-average SM adoption rates, demonstrating the generational gap in SM use.^27,28^

Furthermore, other GPs understood the importance of SM, yet stated that there were other, more pressing concerns in relation to mental health, including other social factors such as alcohol, finances, and home life. It is hypothesised that GPs prioritised these alternative social factors as they have historically been known to affect anxiety levels while SM is a relatively new and complex domain, being popularised in the early 2000s.^29^ Literature provides support for this hypothesis, with GPs often invoking the law of parsimony, in which they opt to diagnose and manage patients based on the simplest explanation of the issue (the alternate social factors), rather than a more complex potential contributor such as SM use.^30^ With medicine generally being a self-regulating profession, it is vital that clinicians are aware of their biases in order that they can provide personalised and patient-focused care.

Finally, regarding barriers, GPs expressed a common concern that the numerous demands on primary care services often limited the time available for their consultations, which, in turn, hindered their ability to thoroughly delve into patient’s subjective experience of anxiety, and comprehensively identify all the underlying factors contributing to their condition, such as SM.

Regarding facilitators, the study found that there was a willingness of GPs to address the impact of SM on young adult anxiety and this could be facilitated through means such as a framework for discussion, greater awareness, being open to discussion, and patient–doctor dynamic.

Additionally, it was suggested that GPs would be more comfortable implementing strategies that came from trusted organisations. While informal implementation of alleviating strategies for SM’s impact on mental health might provide case-by-case relief, mandatory training would have the greatest impact.^31^

General practice is moving towards a model that envisages collaboration with local authorities.^32^ This notion was echoed in our findings, with GPs suggesting that patients may benefit from community-based support groups. This would allow for SM‘s impact on mental health to be addressed sufficiently, as service burden would decrease in the short-term by providing patients with alternative care pathways.

Furthermore, GPs suggested the implementation of a mental health lead, that is, a GP trained to deal with mental health more holistically, including aspects such as SM. Mental health leads could exert an influence on the patients, the GPs, as well as other key organisations to drive a change in attitudes surrounding SM in primary care. Similar benefits have been achieved through the implementation of a mental health nurse-led primary care liaison service, which increased clinical effectiveness, patient centeredness and efficiency; providing evidence for the role of wider workforce involvement in addressing this issue.^33^

Addressing the impact of SM in young adults within primary care is only possible when young adults have a desire to improve their health and receive advice from GPs. GPs suggested that one way in which such conversations could be initiated is through empowering young adults to bring it up in the consultation process. Having confidence and trust within their GPs, leads to better satisfaction, adherence to advice, and symptomatic improvement for patients, all vital in addressing SM’s impact on mental health within primary care.^34^

### Strengths and limitations

This study, to our knowledge, is the first to investigate facilitators and barriers to addressing SM’s impact on anxiety within primary care. The diverse cohort of seven participants, representing various ethnic groups and UK-practice locations, aimed for a broad perspective. The interviews were conducted in an extended format owing to the novel concept. The decision to conclude recruitment after the seventh interview was intentional, as data saturation was achieved, and no new substantial insights were anticipated. However, acknowledging the potential bias from snowball sampling leading to small sample size is essential. The participants, aged 30-57 years, with the majority aged >50 years, may limit the generalisability of findings globally. While more representation from GPs in university practices could enhance insights, the study sample’s diversity and shared views on barriers and strategies strengthen the findings' transferability.

### Comparison with existing literature

There is limited literature exploring the possibility of addressing SM’s impact on anxiety within primary care. However, literature has highlighted the impact that SM has on the mental health of young people.^4–7^ Prior research delving into barriers and facilitators in the improvement of mental health care in primary care also identified common themes such as additional resources, training, and time.^35^

### Implications for research and practice

A framework for questioning was suggested by the GPs to facilitate conversations surrounding SM during mental health consultations. Frameworks have extensively been adopted by doctors to support their clinical practice.^36^

An initial framework, named CALM (Figure 2), has been proposed, drawing on previous research looking at perceptions of young adults,^7^ and each element of the framework has been mapped to address the barriers identified in this study (Supplementary Table S1).^37^ This framework, however, has not been tested yet within primary care or with young adults. The validation and refinement of this framework can only occur through systematic evaluation and input from the target demographic, ensuring its effectiveness in real-world applications.

In conclusion, this research can guide further exploration with a larger GP sample for deeper insights and resource development on consultation strategies about SM. As digital interactions rise,^38^ studying anxiety in the SM context and potential solutions, such as user education,^39^ is crucial. The solutions identified should not be limited to just anxiety management, with a growing body of evidence regarding SM and depression, self-harm, eating disorders, and other forms of poor mental health.^40–43^ While the study delves into primary care, recognising the vital roles of various stakeholders, such as young individuals, psychiatry, university mental health teams, government, and SM companies, is essential. Future research should investigate their facilitators and barriers for holistic, sustainable solutions. As people increasingly spend time on, and interact with SM,^44–46^ future research exploring the potential impact of various SM on people’s health is richly deserving.

## Data

Data are available from the corresponding author on reasonable request.
